# Abnormalities in Structural Covariance of Cortical Gyrification in Parkinson's Disease

**DOI:** 10.3389/fnana.2017.00012

**Published:** 2017-03-07

**Authors:** Jinping Xu, Jiuquan Zhang, Jinlei Zhang, Yue Wang, Yanling Zhang, Jian Wang, Guanglin Li, Qingmao Hu, Yuanchao Zhang

**Affiliations:** ^1^Key Laboratory for Neuroinformation of the Ministry of Education, School of Life Science and Technology, University of Electronic Science and Technology of ChinaChengdu, China; ^2^Institute of Biomedical and Health Engineering, Shenzhen Institutes of Advanced Technology, Chinese Academy of SciencesShenzhen, China; ^3^Department of Radiology, Southwest Hospital, Third Military Medical UniversityChongqing, China; ^4^Department of Neurology, Southwest Hospital, Third Military Medical UniversityChongqing, China; ^5^Key Laboratory of Human-Machine Intelligence Synergy Systems, Shenzhen Institutes of Advanced Technology, Chinese Academy of SciencesShenzhen, China; ^6^Center for Information in Medicine, University of Electronic Science and Technology of ChinaChengdu, China

**Keywords:** Parkinson's disease, structural covariance network, cortical gyrification, brain network, local gyrification index

## Abstract

Although abnormal cortical morphology and connectivity between brain regions (structural covariance) have been reported in Parkinson's disease (PD), the topological organizations of large-scale structural brain networks are still poorly understood. In this study, we investigated large-scale structural brain networks in a sample of 37 PD patients and 34 healthy controls (HC) by assessing the structural covariance of cortical gyrification with local gyrification index (lGI). We demonstrated prominent small-world properties of the structural brain networks for both groups. Compared with the HC group, PD patients showed significantly increased integrated characteristic path length and integrated clustering coefficient, as well as decreased integrated global efficiency in structural brain networks. Distinct distributions of hub regions were identified between the two groups, showing more hub regions in the frontal cortex in PD patients. Moreover, the modular analyses revealed significantly decreased integrated regional efficiency in lateral Fronto-Insula-Temporal module, and increased integrated regional efficiency in Parieto-Temporal module in the PD group as compared to the HC group. In summary, our study demonstrated altered topological properties of structural networks at a global, regional and modular level in PD patients. These findings suggests that the structural networks of PD patients have a suboptimal topological organization, resulting in less effective integration of information between brain regions.

## Introduction

Parkinson's disease (PD) is a progressive neurodegenerative disorder that is characterized by tremor, muscle stiffness or rigidity, slowness of movement or bradykinesia and postural instability (Jankovic, 2008). Although the exact mechanism underlying the pathophysiology of PD is unknown, increasing evidence suggests that it is associated with abnormal cortical morphology and connectivity involving widespread brain regions (Braak and Braak, 2000). Quantitative analysis of the morphological changes of the cerebral cortex provides a potential informative way of uncovering the pathological deviations in PD. Using a surface-based local gyrification index (lGI), our previous study observed significantly reduced gyrification in multiple brain regions in PD patients (Zhang et al., 2014). Although such univariate analysis could highlight the roles played by each brain region in the pathogenesis in PD, it does not allow us to evaluate the interaction or functional integration among brain regions.

Recently, large-scale brain network analysis has been applied to both healthy subjects and diseased populations through interregional correlation of the blood oxygenation level dependent (BOLD) signal (Van Den Heuvel et al., 2008), cortical thickness (Khundrakpam et al., 2013; Zhao et al., 2015), or streamline-based fiber tracking (Gong et al., 2009). These methods not only can provide powerful modes to detect subtle differences in brain organization (He et al., 2007; Bullmore and Sporns, 2009), but also can bring new insights into relevant network parameters that have profound effects on the dynamic performances of a network, such as the local efficiency and global efficiency, against pathological attacks by disease (Bassett and Bullmore, 2006). Based on wavelet correlation, Skidmore and his colleagues (Skidmore et al., 2011) observed reduced global and nodal efficiency in PD patients. By assessing whole-brain intrinsic connectivity, a recent study identified altered topological parameters at a global, intermediate and local level in PD patients (Göttlich et al., 2013). Further, a longitudinal magnetoencephalography (MEG) study (Olde Dubbelink et al., 2014) observed lower local clustering with preserved path length in the delta frequency band in PD patients. All these analyses were conducted in the functional domain, while the structural covariance patterns at the whole brain level are yet to be investigated in PD patients.

Among various structural covariance patterns of the brain, cortical gyrification, a process by which the brain undergoes changes in cortical surface morphology to create sulcal and gyral regions, appears especially relevant to the development of brain as a connected system (Chen et al., 2013). Moreover, alterations in network parameters based on structural covariance have been observed in various psychiatric and neurological disorders (Achard and Bullmore, 2007; Palaniyappan et al., 2015). Quantitative investigations of the structural covariance of cortical gyrification in PD patients might contribute to the understanding of this disorder.

In the current study, large-scale structural brain networks were constructed for 37 PD patients and 34 healthy controls (HC) by assessing the structural covariance of cortical gyrification with lGI. Network topological properties, such as the path length, local efficiency, global efficiency and clustering coefficient, were computed and compared between the two groups. Regional nodal characteristics of brain networks were also assessed to investigate the differences of the hub distribution between the PD and HC groups. Moreover, modularity, one of the main organizing principles in most complex systems (Newman, 2006), was used to identify a set of modules that are structurally or functionally associated with components that perform specific biological functions.

## Materials and methods

### Participants

Initially, 40 PD patients without dementia and 34 HC were recruited consecutively from Southwest Hospital. The diagnosis of PD was according to the UK Parkinson's Disease Society Brain Bank criteria (Hughes et al., 1992). All the participants underwent extensive neurologic, neuropsychologic, and clinical imaging examinations. The participants who had a history of neurologic or psychiatric disease and neurologic sequelae induced by brain trauma were excluded. Movement symptom severity for each side of the body was assessed using the motor examination of the Unified Parkinson's Disease Rating Scale (UPDRS, part III; clinician-scored motor evaluation) following overnight withdrawal from medication and within 1 week postMRI scan examination (Fahn and Elton, 1987). Detailed demographics and clinical status are summarized in Table 1. All protocols were approved by the institutional review board of the Third Military University. All participants signed an informed consent prior to participation in the study.

**Table 1:** **Demographics and clinical data**.

	**PD patients (*n* = 37)**	**Controls (*n* = 34)**	***P*-value**
Age (years)	58.68 ± 13.10	55.59 ± 10.55	0.2805^a^
Gender (male/female)	20/17	12/22	0.1776^b^
Subtype (TD/AR)	13/24		
Duration of illness (years)	3.87 ± 3.10		
Young onset	7		
UPDRS scores	19.17 ± 9.22		
LEDD (mg/day)	325.27 ± 173.42		
MCI	5		

a *The p-value was obtained by a two-tailed two-sample t-test*.

b *The p-value was obtained by a chi-square test*.

*TD, tremor-dominant; AR, akinetic-rigid; UPDRS, Unified Parkinson's Disease Rating Scale; LEDD, levodopa equivalent daily dose; MCI, mild cognitive impairment*.

### MRI data acquisition

Three-dimensional T1-weighted structural MRI scans were obtained on a 3.0 T Siemens Tim Trio whole-body MRI system (Siemens Medical Solutions, Erlangen, Germany) using the volumetric 3D magnetization prepared rapid gradient-echo (MP-RAGE) sequence. Detailed scan parameters were: repetition time = 1,900 ms, echo time = 2.52 ms, slice thickness = 1 mm, no gaps, slices, 176, flip angle = 9°, matrix = 256 × 256, field of view = 256 × 256 mm^2^, and 1 × 1 mm^2^ in-plane resolution on each subject.

### Construction of gyrification-based networks

Firstly, each individual cortical surface was preprocessed using FreeSurfer (http://surfer.nmr.mgh.harvard.edu/) with its standard preprocessing pipelines. Starting from the segmentation of white matter and tessellation of the gray/white matter boundary, an initial surface was obtained after automated topological correction. For quality control, the pial and white matter surfaces were then visually inspected for errors, and edited when necessary according to the FreeSurfer editing manual (https://surfer.nmr.mgh.harvard.edu/fswiki/FreeviewGuide/FreeviewWorkingWithData/FreeviewEditingaRecon). As a result, three PD patients with serious topological defects in the cortical surfaces were excluded. Secondly, the lGI was calculated in three main steps advocated by Schaer et al. (2008) on the basis of an index originally proposed by Zilles et al. (1988). This method provides LGIs, numerical values assigned in a continuous fashion to each vertex of the reconstructed cortical sheet. The LGI of a vertex corresponds to the ratio of the surface area of the folded pial contour (“buried” surface) to the outer contour of the cortex (“visible” surface) included within spherical regions of interest (25 mm radius). Thirdly, the lGI map was parcellated into 68 brain regions with 34 identical regions on each hemisphere (excluding the corpus callosum) using the Desikan-Killiany atlas (http://surfer.nmr.mgh.harvard.edu/fswiki/CorticalParcellation) (Table 2). The average lGI of all vertices that were included in a subregion was taken as the gyrification index value for the corresponding brain regions. Then, the interregional correlation matrix *C* = [*c*_*ij*_] (*i, j* = 1, 2,…*N*, here *N* = 68) of each group was obtained by calculating the Pearson's correlation coefficients across individuals between the lGIs of every pair of regions (He et al., 2007). Prior to the correlation analysis, a linear regression was performed at every cortical region to remove the effects of age, gender and intracranial volume. Finally, the correlation matrix of each group was thresholded with a fixed sparsity ranging from 15 to 40% into a binarized matrix *B* = [*b*_*ij*_].

**Table 2:** **Cortical regions include in the Desikan–Killiany atlas performed under FreeSurfer v5.1.0**.

**Region**	**Abbreviations**	**Index (left)**	**Index (right)**
Banks of the superior temporal sulcus	bSTS	1	35
Caudal anterior cingulate	CAR	2	36
Caudal middle frontal gyrus	cMFG	3	37
Cuneus	CUN	4	38
Entorhinal cortex	EC	5	39
Fusiform gyrus	FG	6	40
Inferior parietal gyrus	IPG	7	41
Inferior temporal gyrus	ITG	8	42
Isthmus cingulate	IC	9	43
Lateral occipital gyrus	LOG	10	44
Lateral orbitofrontal gyrus	LFGor	11	45
Lingual gyrus	LG	12	46
Medial orbitofrontal gyrus	MFGor	13	47
Middle temporal gyrus	MTG	14	48
Parahippocampal gyrus	ParaHIPP	15	49
Paracentral gyrus	ParaCG	16	50
Pars opercularis	pOPER	17	51
Pars orbitalis	pORB	18	52
Pars triangularis	pTRI	19	53
Pericalcarine cortex	PeriCAL	20	54
Postcentral gyrus	PostCG	21	55
Posterior cingulate	PCC	22	56
Precentral gyrus	PreCG	23	57
Precuneus	PreCUN	24	58
Rostral anterior cingulate	RAC	25	59
Rostral middle frontal gyrus	rMFG	26	60
Superior frontal gyrus	SFG	27	61
Superior parietal gyrus	SPG	28	62
Superior temporal gyrus	STG	29	63
Supramarginal gyrus	SupraMG	30	64
Frontal pole	Fpole	31	65
Temporal pole	Tpole	32	66
Transverse temporal gyrus	TTG	33	67
Insula	INS	34	68

### Graph theoretical analysis

To investigate the topological properties of the structural networks obtained from the HC and PD groups, we used a number of network properties: path length (*L*_*p*_), local efficiency (*E*_*local*_), global efficiency (*E*_*global*_), clustering coefficient (*C*_*p*_), and small-world index (*SWI*).

(1) The shortest path length of a node in the network *G* (N, E) is defined as:

(1) $$\begin{array}{l} L_{i}=\frac{1}{N-1}\underset{i\neq j\in G}{\sum }d_{ij}, \end{array}$$

in which *d*_*ij*_ is the shortest absolute path length between the *i* and *j* nodes. *L*_*p*_ is the average of the shortest path length between the nodes:

(2) $$\begin{array}{l} L_{p}=\frac{1}{N}\underset{i\in G}{\sum }L_{i}. \end{array}$$

which quantifies the extent of average connectivity or the overall routing efficiency of the network (Achard and Bullmore, 2007).

(2) The global efficiencyof G (N, E) is defined as:

(3) $$\begin{array}{l} E_{Global}\left(G\right)=\frac{1}{N\left(N-1\right)}\underset{i\neq j\in G}{\sum }\frac{1}{d_{ij}}, \end{array}$$

The reflecting the global efficiency of parallel information transfer in the network (Achard and Bullmore, 2007), where *d*_*ij*_ is the shortest path length between nodes *i* and *j* in *G*.

(3) The local efficiency of G (N, E) is defined as:

(4) $$\begin{array}{l} E_{Local}\left(G\right)=\frac{1}{N}\underset{i\in G}{\sum }E_{Global}\left(G_{i}\right), \end{array}$$

where *E*_*Global*_ (*G*_*i*_) is the global efficiency of *G*_*i*_, the sub-graph of the neighbors of node *i*, which can be understood as a measure of the fault tolerance of the network, indicating how well each subgraph exchanges information when the index node is eliminated (Achard and Bullmore, 2007).

(4) Clustering coefficient of a node *i* is defined as the number of existing links divided by the number of all possible links among the neighbors of a node:

(5) $$\begin{array}{l} Ci=\frac{2E_{i}}{K_{i}\left(K_{i}-1\right)}, \end{array}$$

where *K*_*i*_ is the number of connections to node *i, E*_*i*_ is the number of existing connections among the neighbors. The clustering coefficient of a network is the average of the clustering coefficient of all nodes:

(6) $$\begin{array}{l} C_{p}=\frac{1}{N}\underset{i\in G}{\sum }C_{i}, \end{array}$$

which is a measure of the extent of local cliquishness or local efficiency of information transfer of a network (Latora and Marchiori, 2001).

(6) *SWI* of a network is defined as:

(7) SWI=CpCnullLpLnull,

where *C*_*null*_ and *L*_*null*_ are respectively the clustering coefficient and the path length of a random network which has the same number of nodes, edges and degree distribution as the gyrification-based network.

To have a summary metric and a comparative measure that can be used for all the network groups, we used the normalized integrals of the network parameters:

(8) $$\frac{\int_{a}^{b}L_{p}}{b-a},\frac{\int_{a}^{b}E_{Global}}{b-a},\frac{\int_{a}^{b}E_{Local}}{b-a},\frac{\int_{a}^{b}C_{p}}{b-a},\frac{\int_{a}^{b}SWI}{b-a},$$

where *a* and *b* are respectively the lower and upper limits of sparsity (here, *a* = 15 and *b* = 40).

### Network statistical analysis

For group comparison of network parameters, we generated 1,000 bootstrap samples (with replacement) from each group (Markus and Groenen, 1998) and computed a gyrification correlation matrix for each sample. The *L*_*p*_, *E*_*local*_, *E*_*global*_, and *C*_*p*_ were computed from the correlation matrix of each bootstrap sample, over the range of sparsity thresholds and their summary metrics. Normalized integrals of the network parameters were used to compare between the HC and PD groups. The distributions of the 1,000 summary graph metrics were checked for normality. Then two sample *t*-tests were used to assess the significant difference of the integrated *L*_*p*_, integrated *E*_*local*_, integrated *E*_*global*_, and integrated *C*_*p*_ between the two groups.

### Global hubs in the network

In this study, we examined the nodal characteristics of the cortical gyrification network using “betweenness centrality” in the HC and PD groups. The betweenness of a node *i* is defined as the number of shortest paths between any two nodes that run through node *i*, and is denoted as *bc*_*i*_. We defined the normalized betweenness as $BC_{i}{=}^{bc_{i}}{/}_{\bar{bc_{i}}}$, where $\bar{bc_{i}}$ was the average betweenness of all the nodes. Then, we averaged the normalized betweenness across the range of sparsity. Regions with a higher value of $\bar{BC_{i}}$ (> mean + SD) were identified as the global hubs in the brain network.

### Modularity analysis

A module can be generally defined as a subset of nodes in the graph that are more densely connected to other nodes in the same module than to nodes outside the module (Radicchi et al., 2004). The Newman's optimization algorithm can detect the optimum number of modules by giving the highest possible modularity value, which is defined as the difference between the numbers of intra-modular links in a given network and the number of inter-modular links that will be seen in a random network for the same numbers of modules. To investigate the abnormal interactions between and within these modules, we defined the inter- and intra-modular connectivity respectively as the mean correlation coefficient of all pairs of nodes in two different modules and one single module. The mean correlation coefficient was then converted to *z* values using Fisher's *r*-to-*z* transformation. Furthermore, we also computed the average integrated regional efficiency of each module using the bootstrap samples and compared them between the two groups.

## Results

### Gyrification-based networks

**Figure 1:**
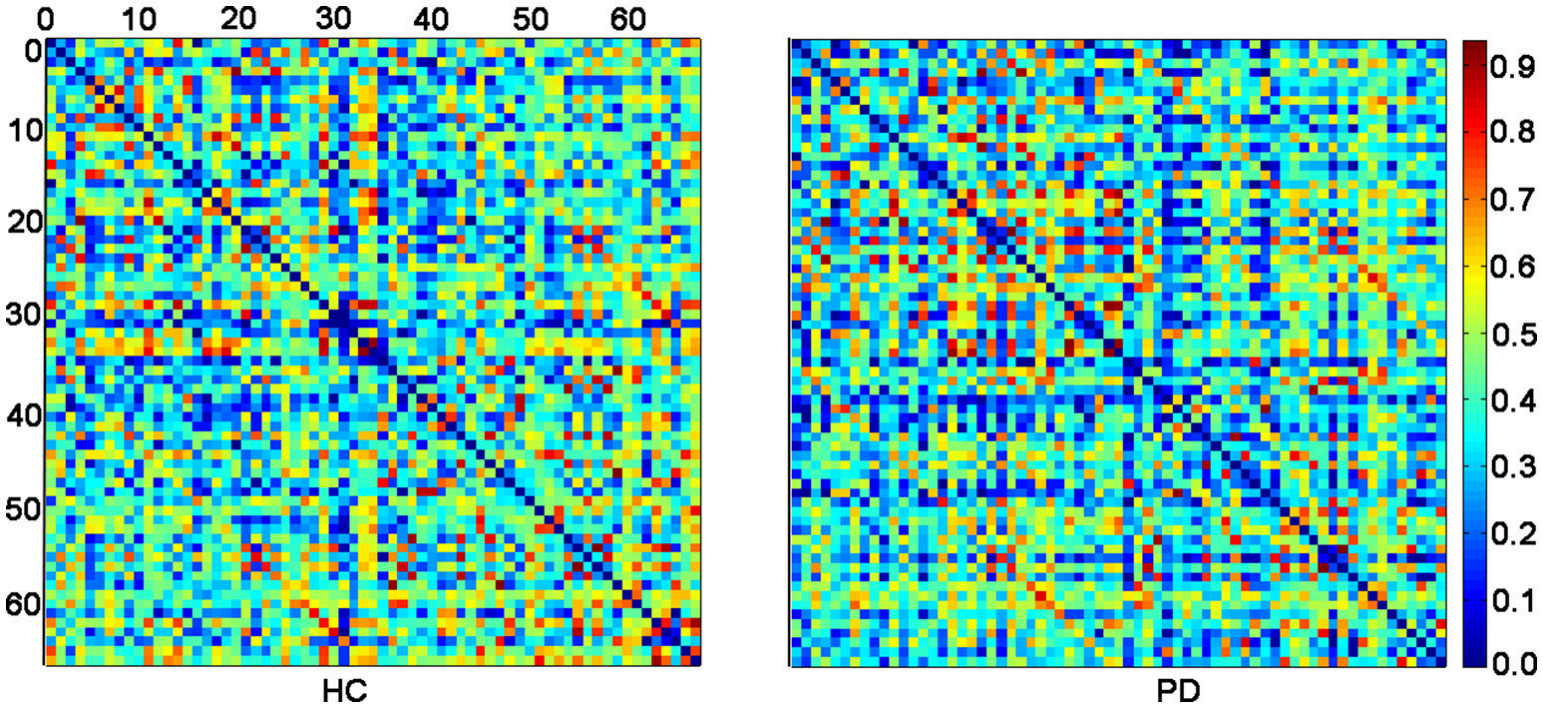
**The inter-regional correlation matrices for HC and PD groups**. The connectivity matrix shows the Pearson correlation coefficient between any two nodes of the network. The nodes are numbered according to Table 2 for better overview. The color bar represents the absolute value of the Pearson correlation coefficient, which ranged from 0 (blue) to 1 (red).

**Figure 2:**
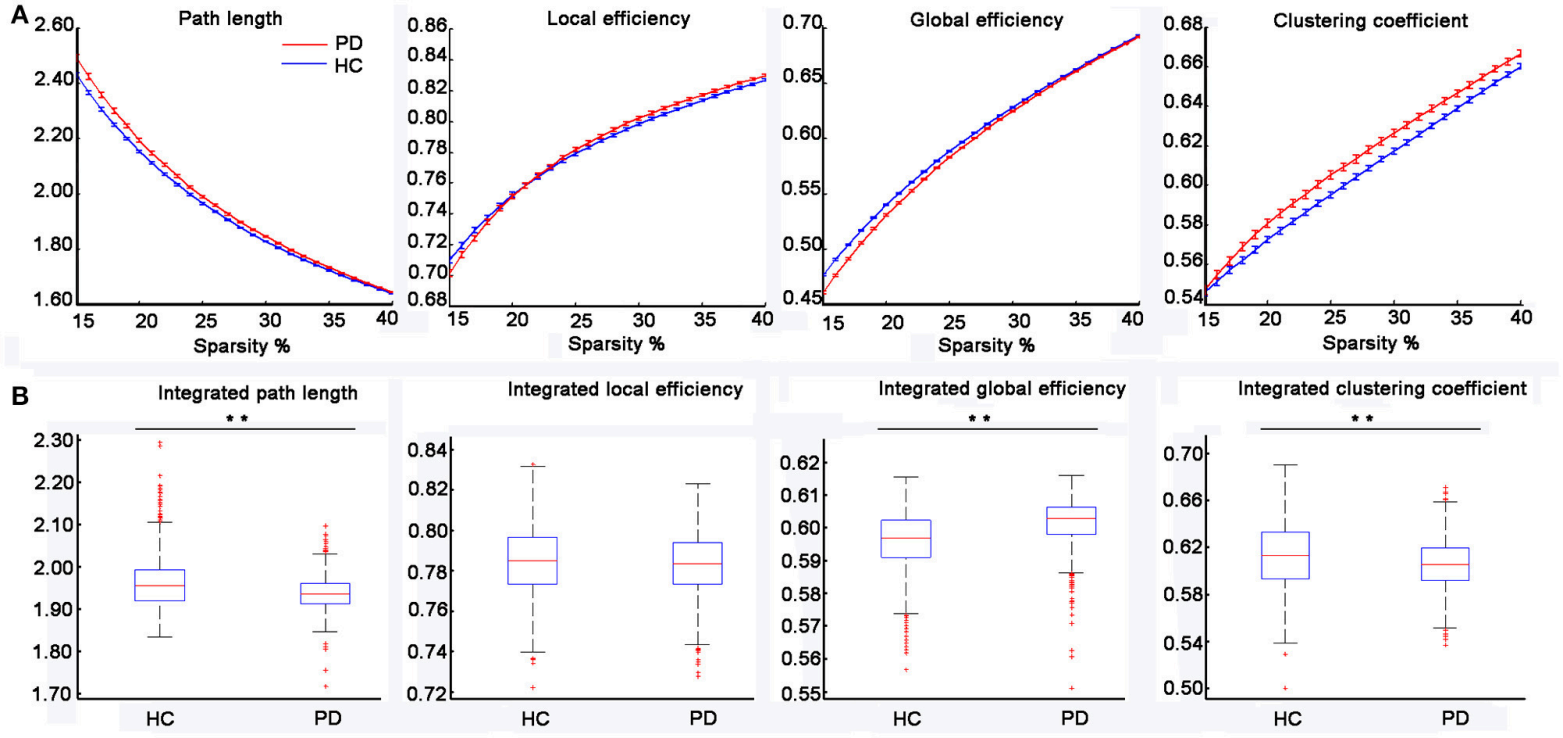
**Changes in the topological parameters of the structural network in PD patients. (A)** The path length, local efficiency, global efficiency and clustering coefficient for the HC and PD groups as a function of sparsity (15–40%). **(B)** Statistical comparisons of the graph metrics, namely the integrated path length, integrated local efficiency, integrated global efficiency and integrated clustering coefficient for a range of sparsity (15–40%) using 1,000 bootstrap samples. The distributions of the 1,000 summary graph metrics were checked for normality and two-sample *t*-test was used to examine the significant difference of a summary graph metric between the two groups (i.e., *p* < 0.05/4 = 0.0125 with a strict Bonferroni correction, ^**^represents significant differences between the two groups).

Figure 1 displayed the gyrification correlation matrices of the HC and PD groups. The overall global topological parameters, namely *L*_*p*_, *E*_*local*_, *E*_*global*_, and *C*_*p*_ of the structural networks of the two groups are shown in Figure 2 as a function of sparsity. The cortical networks of both groups showed prominent small-world indices (SWIs, average SWI across the range of sparsity for *HC* = 1.6408 and *PD* = 1.6924). Moreover, we found significantly increased integrated path length and integrated clustering coefficient, as well as decreased integrated global efficiency in PD patients as compared to the HC group (two sample *t*-test at *p* < 0.05/4 = 0.0125 with a strict Bonferroni correction).

### The distributions of global hubs

**Figure 3:**
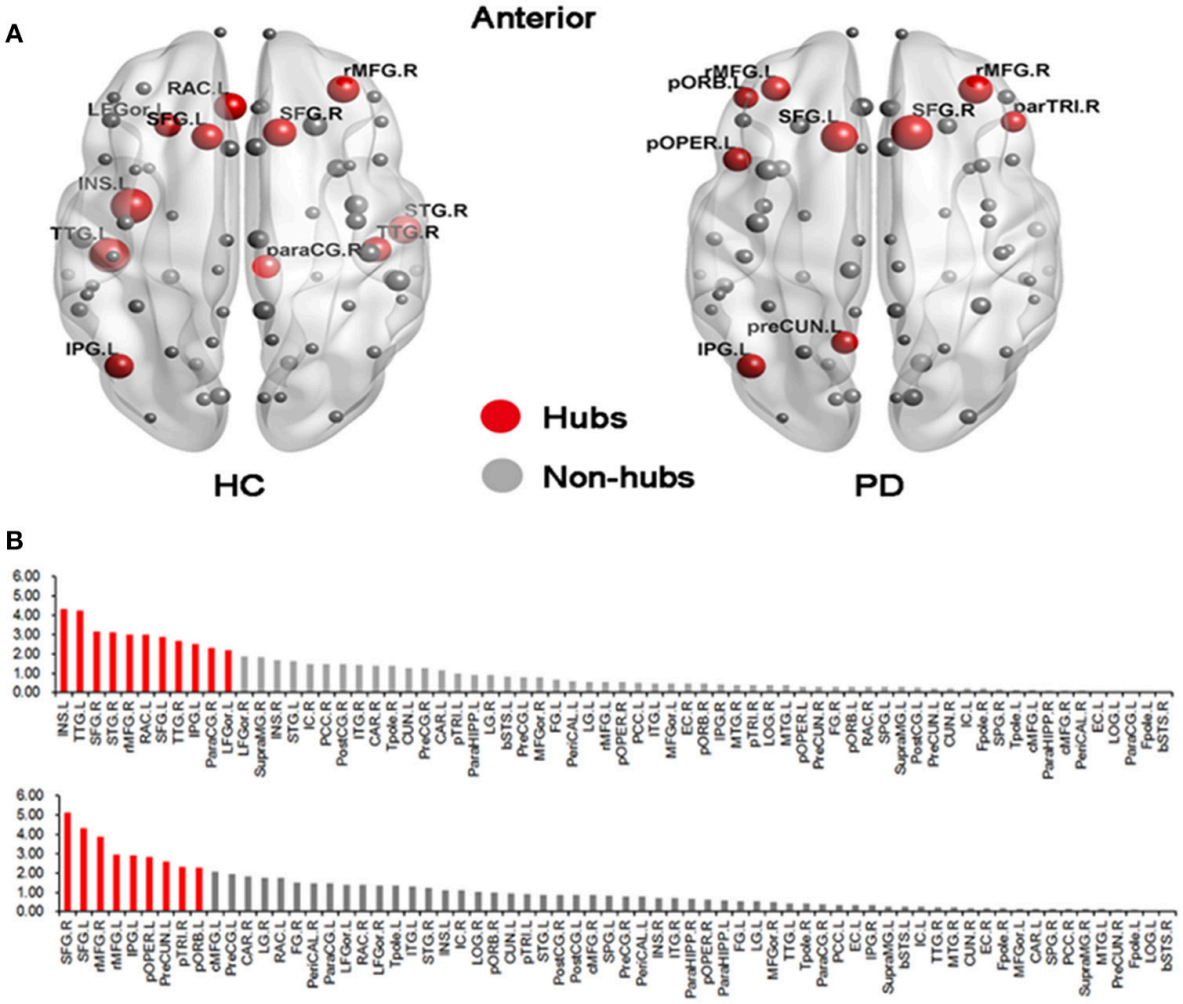
**Distributions of hub regions in the HC and PD groups. (A)** Graphical representation of gyrification networks in the HC and PD groups, visualized using BrainNetviewer (http://www.nitrc.org/projects/bnv). The size of the nodes is proportional to the nodal betweenness in the networks. **(B)** Bar plot of betweenness centrality in all regions (upper: HC; bottom: PD). The red regions were hubs of the network and gray ones were non-hubs. All abbreviations were listed in Table 2.

We found different global hub distributions of the cortical networks for the two groups (Figure 3). In the HC group, 11 regions were identified as hubs because of large values in the normalized betweenness $\bar{BC_{i}}$ (> mean + SD), including 5 regions in the frontal cortex (the left rostral anterior cingulate, the left lateral orbitofrontal gyrus, the bilateral superior frontal gyrus, and the right rostral middle frontal gyrus), 3 regions in the temporal cortex (the bilateral transverse temporal gyrus and the right superior temporal gyrus), 2 regions in the parietal cortex (the right paracentral gyrus and the left inferior parietal gyrus), and 1 subcortical regions (the left insula). However, in the PD group, 7 of 9 hub regions were located in the frontal cortex (the bilateral rostral middle frontal gyrus, the left pars orbitalis, the left pars opercularis, the bilateral superior frontal gyrus, and the right pars triangularis), and the other 2 regions in the parietal cortex (the left precuneus and the left inferior parietal gyrus). The bilateral superior frontal gyrus and the left inferior parietal gyrus were identified in both groups.

### Modular difference

**Figure 4:**
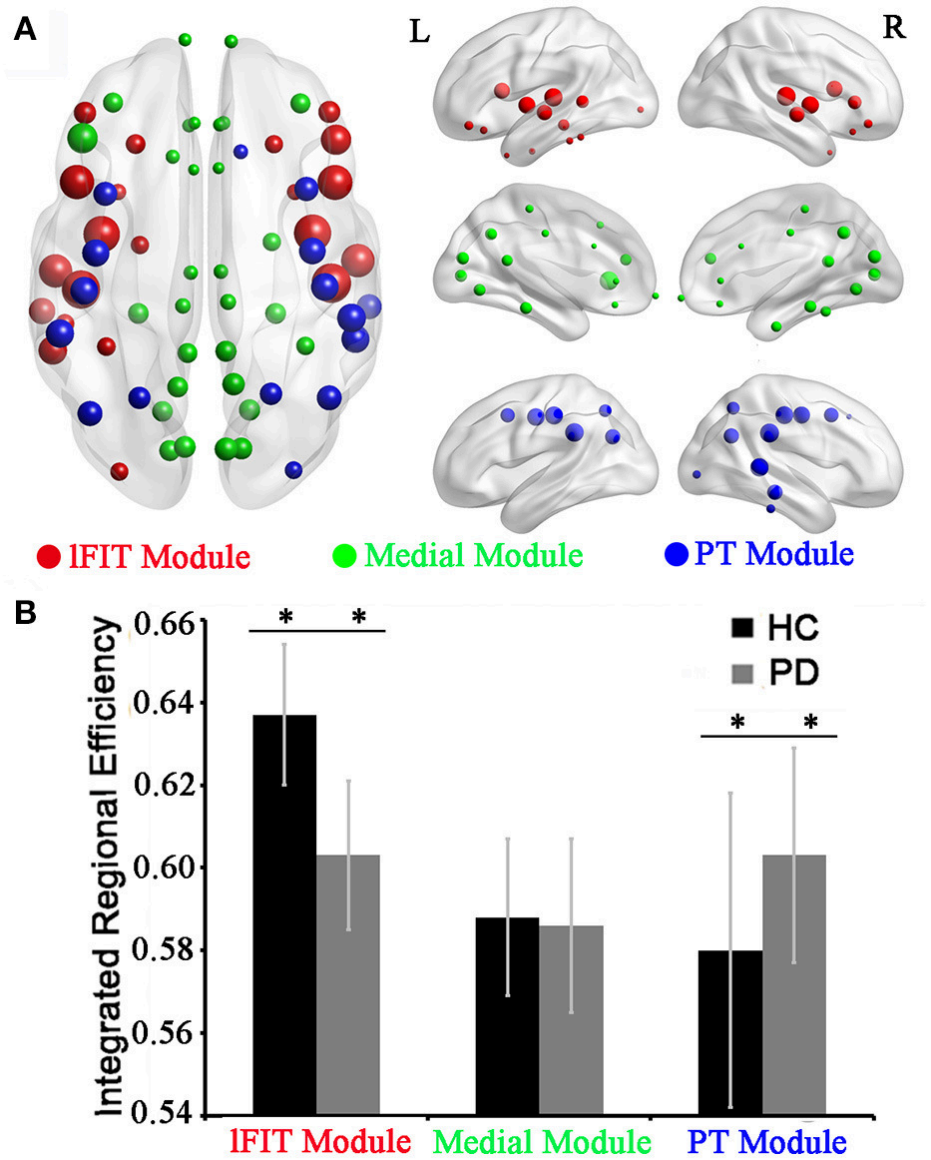
**Modular differences of regional efficiency in the HC and PD groups. (A)** Graphical representation of modular distributions in the HC group, visualized using BrainNetviewer. Using Newman's module detection algorithm, we identified 3 modules from the structural network of the HC group, which were coded separately (Red, lFIT module; Green, Medial module; and blue, PT module). The size of the nodes is proportional to the nodal betweenness in the networks. **(B)** Statistical comparisons of regional efficiency of brain divisions in the HC and PD groups using two-sample *t*-tests with a strict Bonferroni correction (i.e., *p* = 0.05/3 ≈ 0.016 as threshold, ^**^represents significant differences between the two groups). Abbreviations: medial module for midline structures (Medial module), lateral Fronto-Insula-Temporal module (lFIT module), and Parieto-Temporal module (PT module).

In the HC group, three optimal modules were identified including module 1 (21 regions), designated as the lateral Fronto-Insula-Temporal module (lFIT module), module 2 (30 regions), designated as the medial module for midline structures (Medial module), and module 3 (17 regions), designated as the Parieto-Temporal module (PT module). The detailed modular structures of the structural network of the HC group were shown in Figure 4A. Compared with the HC group, no significant inter-modular or intra-modular connectivity difference was observed in PD patients. Whereas PD patients showed significantly decreased integrated regional efficiency in lFIT module and increased integrated regional efficiency in PT module as compared to the HC group (Figure 4B, two sample *t*-test at *p* = 0.05/3 ≈ 0.016 with a strict Bonferroni correction).

## Discussion

By assessing interregional correlation of lGI, we constructed the structural networks for 37 PD patients and 34 HC and performed a comprehensive investigation of the structural networks of the two groups, which revealed altered network parameters at a global, regional and modular level in PD patients.

A small-world network is characterized by a high local clustering of connections between neighboring nodes and short path lengths between any pair of nodes, which reflects high segregation and integration efficiency. To date, the small-world attribute seems to be convergent evidence from methodologically disparate studies in multiple functional and structural human brain networks (Bassett et al., 2008; Liu et al., 2008; Li et al., 2009; Zhang et al., 2012; Palaniyappan et al., 2015). In line with previous studies, small-world attributes were observed in both the HC and PD groups in this study. The presence of the small-world properties in the PD group suggested that abnormalities in the gyrification patterns are subtle and do not affect the basic organization principles of cortical folding. Moreover, our results also supported the common finding that small-world topology is a fundamental principle of the structural and functional organization of complex brain networks (Bassett and Bullmore, 2006; Gong et al., 2009).

Notably, several previous studies (Skidmore et al., 2011; Göttlich et al., 2013; Olde Dubbelink et al., 2014) have documented altered brain network properties in PD patients. Our work further extends previous studies by showing that the combination of graph theory with gyrification analysis can be used to investigate differences in network properties between the HC and PD groups. As expected, the present study observed significantly decreased integrated global efficiency in PD patients. This finding was in line with a previous fMRI study (Skidmore et al., 2011) which found lower global efficiency of the brain networks of PD patients as compared to that of the control subjects. Indeed, brain networks with high global efficiency or low path length assure effective integrity or rapid transfer of information between and across remote regions, which were believed to constitute the basis of cognitive process (Sporns and Zwi, 2004). Thus, our finding of decreased integrated global efficiency in PD patients indicated that structural brain networks of PD patients take on a less optimal configuration during cognitive processes, which might be the structural substrates underlying the cognitive dysfunction of this disorder.

Despite the absence of prominent alterations in the integrated local efficiency, both the integrated clustering coefficient and integrated path length were higher in the PD group as compared to the HC group. These findings were similar to the results of a previous resting-state brain network analysis (Göttlich et al., 2013) which showed higher clustering coefficient and path length at a low sparsity value of 0.2 in PD patients. The longer characteristic path length, which indicates a lower speed of information transferring, combined with the higher clustering coefficient, which indicates a stronger local specialization (Zhang et al., 2012), in the gyrification covariance networks of PD patients suggests that the normal balance between local specialization and global integration was disturbed (Sporns et al., 2000; Zhang et al., 2012), rendering their networks more in favor of a regular configuration.

Using betweenness centrality, we identified the hub regions of the cortical networks for the two groups. In the HC group, the hub regions were distributed in the frontal cortex, the temporal cortex, the parietal cortex, and the sub-cortex. However, in the PD group, the hub regions were mostly located in the frontal cortex and the parietal cortex. Although the identified hub regions varied between the two groups, most of these regions were found to show high regional efficiency or betweenness centrality in previous studies (Shu et al., 2009; Wu et al., 2012; Göttlich et al., 2013). The discrepancies of identified hub regions among studies could be due to the different neuroimaging modalities, subjects' characteristics and computational methods. Moreover, the higher betweenness centrality, particularly in regions of the frontal cortex in PD patients, suggested a higher importance of the frontal regions or their connections for information integration. Given that the frontal cortex was mainly involved in high-order cognitive functions, it is tempting to speculate that higher betweenness centrality in these frontal regions may represent a compensatory mechanism to maintain normal cognitive functions.

The analysis of network modules allowed us to obtain deeper insights into the global network properties. In this study, we identified three modules in the HC group including the lFIT module, the medial module, and the PT module. Our modular distributions were similar to a previous gyrification study showing that the three modules were hierarchically gathered according to the distribution of the lGI measurements (Schaer et al., 2008), that is, the lowest (Medial Module), the middle (PT module), and the highest (lFIT module). Considering the altered integrated regional efficiency in the lFIT and PT modules in the PD patients, we may conclude that the more complex the gyrification of the module is, the more easily it tends to be affected. Moreover, most homotopic regions of the two hemispheres were observed to belong to the same module, which may indicate strong interhemispheric coupling according to the gyrification-based networks.

Several limitations should be mentioned. Firstly, most of the PD patients were given dopaminergic medications. Studies of drug-naïve individuals to exclude the effects of dopaminergic medications on the network topological characteristics are warranted. Secondly, the gyrification covariance networks only measure the anatomical connectivity patterns indirectly compared with the anatomical networks. However, with the relatively low computational load and simple definition connections, the gyrification covariance networks are more practical for revealing the anatomical connectivity patterns of the human brain. Finally, since the nature of structural covariance network does not allow for the extraction of the network parameters at an individual level, we cannot examine their correlations with the clinical scores of PD patients. Future diffusion-based network analyses with the availability of individual-level network parameters are needed to investigate the relationship between them.

In summary, we compared the topological properties of the structural cortical networks inferred from interregional correlation of lGI between PD patients and healthy controls. We revealed altered network properties at a global, regional and modular level in PD patients. These findings suggests that the structural networks of PD patients have a suboptimal topological organization, resulting in less effective integration of information between brain regions.

## Author contributions

JX, JLZ, and YW acquired and analyzed data. JZ, JW, YZ and YLZ conceived this study and designed experiments. JX wrote the article with help of GL and QH. All authors were involved in data interpretation and critically revising the manuscript.

## Funding

This work was supported by National Program on Key Basic Research Project (No. 2013CB733800, 2013CB733803), the Key Joint Program of National Natural Science Foundation and Guangdong Province (U1201257), Guangdong Innovative Research Team Program (No. 201001D0104648280), National Natural Science Foundation (No. 61671440, 61135004), and Plan A of Science and Technology Support Program from Science and Technology Department of Sichuan Province (Grand no. 2014SZ0014).

### Conflict of interest statement

The authors declare that the research was conducted in the absence of any commercial or financial relationships that could be construed as a potential conflict of interest.
